# Application of nanotechnology to cancer radiotherapy

**DOI:** 10.1186/s12645-016-0024-7

**Published:** 2016-12-19

**Authors:** Yu Mi, Zhiying Shao, Johnny Vang, Orit Kaidar-Person, Andrew Z. Wang

**Affiliations:** 1 Laboratory of Nano- and Translational Medicine, Lineberger Comprehensive Cancer Center, Carolina Center for Cancer Nanotechnology Excellence, Carolina Institute of Nanomedicine, University of North Carolina at Chapel Hill, Chapel Hill, NC 27599 USA; 2 Department of Radiation Oncology, Lineberger Comprehensive Cancer Center, University of North Carolina at Chapel Hill, Chapel Hill, NC 27599 USA; 3 Jiangsu Center for the Collaboration and Innovation of Cancer Biotherapy, Cancer Institute, Xuzhou Medical College, Xuzhou, China

**Keywords:** Cancer nanotechnology, Radiotherapy, Radioisotope, Radiosensitizer, Combination therapy, Image-guided radiotherapy

## Abstract

Radiotherapy has been an integral treatment modality for cancer. The field arose from and progressed through innovations in physics, engineering, and biology. The evolution of radiation oncology will rely on the continued adoption of advances from other fields. A new area of science that possesses the ability to impact radiation oncology is nanomedicine. Materials on the nanoscale provide many unique properties such as enhanced permeability and retention effect and superparamagnetism that are well suited for applications in radiation oncology. In this review, we will provide a comprehensive summary on how nanotechnology can improve cancer radiotherapy in aspects of treatment delivery and monitoring as well as diagnosis.

## Background

Radiotherapy is one of the most common and effective cancer treatment modalities (Barcellos-Hoff et al. 2005; Bernier et al. 2004). The field began when the Nobel Prize winner Marie Curie discovered radioactivity and its effects on human cells. Ionizing radiation is utilized as a therapeutic approach because it can generate various DNA damage and induce cellular death in target locations (clinical and/or subclinical lesions) (Jackson and Bartek 2009). Since cancer cells divide in an unregulated manner, they are more susceptible and prone to radiation-induced DNA damage (Baskar et al. 2012). Today, more than 60% of cancer patients receive radiotherapy during their anti-cancer treatment (Schaue and McBride 2015), which is applied through various techniques, including external beam (electrons, protons, photons) and brachytherapy (internal radioactive source). Its mode of application depends on the clinical indications.

Innovative technologies that allow for real-time imaging and better dose distribution have significantly improved the therapeutic ratio of radiotherapy. However, challenges remain. Many cancers, such as pancreatic cancer and glioblastoma, are relatively resistant to radiotherapy. There is a need to further improve therapeutic efficacy of radiotherapy in these less radioresponsive tumors. Another challenge is normal tissue toxicity. Chemoradiotherapy, the concurrent administration of chemotherapy and radiotherapy, is part of the standard of care and curative treatment for many cancers. However, the combination treatment also significantly increases toxicity. For example, chemoradiotherapy in lung cancer can carry mortality risk of approximately 5%, which is higher than either chemotherapy or radiotherapy alone (Minami-Shimmyo et al. 2012). Thus, there is also strong interest in novel approaches to reduce treatment toxicity of radiotherapy.

One potential approach to address these challenges is to utilize nanotechnology. The concept arose from unique chemical and physical properties of nanomaterials that are different from molecular or bulky materials. For example, gold nanoparticles show surface plasmon resonance effect and photothermal effect; while gold nanoclusters present fluorescence in visible region. Furthermore, the large surface area of nanomaterials makes them modifiable for high stability, biocompatibility, and interaction with certain cells. They give a solution for many old challenges, especially in biomedical area, such as transportation of drugs in physiological environment or imaging for diagnosis. In oncology, the advantage of using nanosized therapeutic agents is that they have prolonged circulation period in the bloodstream which allows them to reach the target tissue more efficiently. More specifically, considerable carriers reduce the penetration capability to normal tissue and permit passive targeting of the cancerous tissue by exploiting the characteristic features of tumor biology, i.e., disturbed blood vessels with high permeability. The disturbed tumor’s vasculature allows the nanocarriers to easily infiltrate the tumor and the disturbed lymphatics within the tumor. Once inside the cancer cell, the nanosized therapeutic agent’s distinctive capabilities allow accumulation and retention of these agents within the tumor for an extensive amount of time. This aggregation of the nanocarriers inside the tumor is known as the enhanced permeability and retention (EPR) effect.

Not only is this mechanism applicable for chemotherapy and other forms of systemic anti-cancer agents, but the use of nanocarriers will also improve radioisotopes delivery to tumors (Li 2014). In this review, we will discuss how nanotechnology can influence the field of radiotherapy with regard to radiosensitization, the use of radioisotopes, imaging, and monitoring of radiotherapy.

## Applications of nanotechnology to cancer radiotherapy

### Improving radioisotope delivery through nanomedicine

The use of radioisotopes (radionuclide) in clinical practice is well established. Radioisotopes emit energy from the nucleus and generate ionized atoms and free radicals to induce single strand cleavages in DNA. Radioisotopes applied in the clinical oncology include beta-emitters, like ^186^Re, ^188^Re, ^166^Ho, ^89^Sr, ^32^P, and ^90^Y, as well as alpha-emitters, like ^225^Ac, ^211^At, and ^213^Bi (Hamoudeh et al. 2008). When used in vivo, beta-emitters have profound tissue penetration (20–130 mm) but low linear energy transfer, whereas alpha-emitters have limited penetration (50–80 μm) but a short half-life and the ability to inflict more damage to the cells.

There are different mechanisms of how the human body eliminates radioisotopes. Many of the radioisotopes undergo rapid clearance by the kidney. In particular, renal clearance is size dependent, for which size smaller than 5 nm will be excreted rapidly. Radioisotopes as small molecules suffer short circulation time in blood and are unable to achieve therapeutic effect. Another possible elimination process of the radioisotopes is by opsonization, which is an immune process where macromolecules are cleared by the mononuclear phagocyte system (MPS).

However, through loading or conjugating of the nanocarriers, radioisotopes are able to escape from these biological elimination mechanisms. For example, the physical half-life of ^89^Sr is 50.5 days, but it is cleared from plasma with an average half-life of 47 h. Nanoparticles such as liposomes, micelles, or polymeric complex are usually more than 10 nm, which greatly decreases the renal clearance and increases their half-life in blood due to the distinct pharmacokinetic properties and the increased size effect (Brigger et al. 2002; Davis et al. 2008; Feng et al. 2007; Kim et al. 2010). Also, the nanocarriers can prevent opsonization through PEGylation. The presence of polyethylene glycol (PEG) on the surface of nanoparticles produces steric hindrance, which prevents the adsorption of opsonins. This particular characteristic of nanocarriers helps prolong the half-life of radiotherapeutic agents in blood. In a tumor-bearing mice model, the half-lives of ^111^In- and ^177^Lu- PEGylated liposomes in blood were 10.2 and 11.5 h, respectively; whereas the half-life of ^111^In-DTPA in blood was extremely short as no longer than 2 h (Wang et al. 2006).

In addition to the enhancement of circulatory half-life by the nanoparticles, the abnormal vasculatures in tumor may also help to extend the retention time of radiotherapeutics through the EPR effect. The abnormal tumor vasculatures possess aberrant branching components and leaky arterial walls, resulting from rapid proliferation of endothelial cells and a decrease in the number of pericytes. These abnormal vessels allow macromolecules, like nanoparticles, to easily penetrate the tumor via the circulatory system. Since the quick proliferation of tumor cells disrupts lymphatic vessels and makes them inefficient in drainage, the macromolecules that successfully perforate the tumor will be conserved inside the tumor with enhanced retention time. This is a perfect example of the EPR effect and also becoming a golden standard in drug delivery (Fang et al. 2011; Maeda et al. 2000). For instance, Doxil, a PEGylated liposomal formulation of doxorubicin, is a nano-drug approved by Food and Drug Administration (FDA), showing a much slower clearance rate as 0.1 L/h compared with 45 L/h for free doxorubicin. Its AUC after a dose of 50 mg/m^2^ is approximately 300-fold greater than that with free drug. Furthermore, considerable levels of doxorubicin are detected in both tumor cells and tumor interstitial fluids after Doxil administration. Moreover, the peak of drug concentration in tumors appears between 3 and 7 days post administration of Doxil, which reveals a much longer exposure time and a more enhanced concentration in tumors than that after the administration of free doxorubicin (Barenholz 2012).

Radioisotope-labeled nanoparticles have been developed to increase tumor accumulation and reduce undesired biodistribution. Li et al. applied the beta-emitter ^64^Cu-labeled copper sulfide nanoparticles to suppress breast cancer. More than 90% of the nanoparticles were restricted in the tumor 24 h after the intratumoral injection. This radioisotope-labeled nanoparticle showed no obvious side effect, and once combined with photodynamic therapy, it helped to prolong the survival time of 4T1 bearing mice to 7.6 times longer than the control group and further reduced lung metastasis as well (Zhou et al. 2015). Another example involved 50-nm lipid nanocapsules loaded with a lipophilic complex of ^188^Re for internal radiotherapy of glioblastoma. The nanocapsules ensured maximum distribution of ^188^Re within the brain 96 h after injection, compared with the solution of ^188^Re-perrhenate. Therefore, it led to a noteworthy survival advantage in rat glioma models (Vanpouille-Box et al. 2011). Shi et al. synthesized generation five dendrimers with NHAc-HPAO-PEG-FA and conjugated it with ^131^I. Due to the modified folate ligand, the radioactive ^131^I-labeled multifunctional dendrimers can be applied for single-photon emission computed tomography (SPECT) imaging and radiotherapy. The in vivo experiments demonstrated that the relative C6 xenografted tumor volume was only 8.78 times larger than the original one after 21 days, compared with 26.56 times for the control group (Zhu et al. 2015).

### Improving radiosensitizer delivery through nanomedicine

**Fig. 1:**
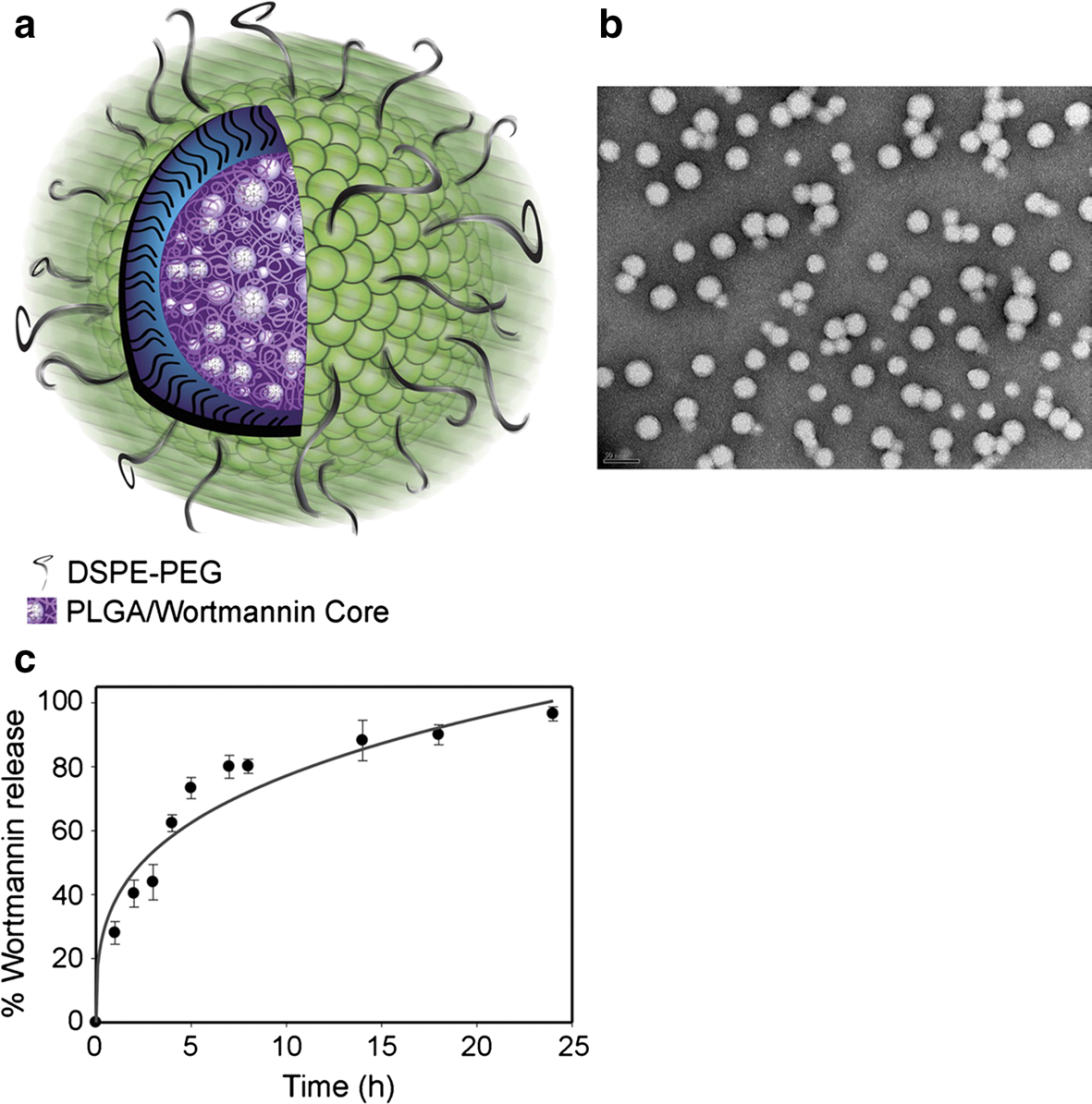
Characterization of NP Wtmn. **a** Cartoon of NP Wtmn depicting a PLGA core containing Wtmn surrounded by a lipid monolayer (*green head groups*) and a PEG shell. **b** TEM image of NP Wtmn. **c** Release profile of NP Wtmn in PBS at 37 °C. *Error bars* correspond to SD of three separate sample preparations with duplicate samples per data point (Karve et al. 2012)

Nanoparticles formulations of known radiosensitizers can improve the delivery of these agents to tumor sites. For example, wortmannin is an inhibitor of phosphatidylinositol 3′ kinases and phosphatidylinositol 3′ kinase-related kinases such as DNA-dependent protein kinases. Preclinical results have shown that it is an effective radiosensitizer. However, its clinical application is limited by poor solubility, low stability, and high toxicity. Formulation of wortmannin with nanoparticles, which is composed of a DSPE-PEG lipid shell and a PLGA polymer core, solved these problems (Fig. 1). The nanoradiosensitizer was demonstrated to be more effective than 5-FU on mice bearing KB cell xenografts and its MTD was three to five times greater than that of wortmannin (Karve et al. 2012). The same strategy was also used for DNA double-strand repair inhibitors, such as histone deacetylase inhibitor, which is an effective radiosensitizer to a variety of solid malignancies such as colorectal cancer and prostate cancer. The inhibitor enhances the response of tumor cells to radiation through the prolongation of γ-H2AX foci. However, it is inefficient at sustaining inhibition of DNA repair and highly toxic. Through encapsulation of nanoparticles, the inhibitors were released controllably for a durable effect. Conjointly, the radiosensitizers in the nano-formulation accumulated in tumors and had low distribution in normal tissue (Tian et al. 2015; Wang et al. 2015).

In addition to the use of drug-loaded polymeric nanoparticles as radiosensitizers, some nanomaterials with high atomic numbers (Z) also have the potential to become radiosensitizers because the dose absorbed by any tissue is related to the Z^2^ of the material. For example, gold (*Z* = 79) nanoparticles are the most broadly used high *Z* nanomaterials for radiosensitizers. Xie et al. reported the application of ultrasmall glutathione-coated Au_29-43_(SG)_27-37_ nanoclusters as radiosensitizers. The nanosensitizers had high tumor uptake of about 8.1% ID/g at 24-h post injection. The inhibition of tumor by irradiation was significantly improved when the gold nanoclusters were administered. Meanwhile, the damage to normal tissues was negligible (Zhang et al. 2015). Gadolinium (*Z* = 64)-based nanoparticles are another type of commonly used radiosensitizers. In one study, Gd-based nanoparticles were used, with 250 kV photon irradiation, to kill SQ20B cells for increased DNA breaks and shortened G2/M phase blockage. In a SQ20B tumor-bearing mouse model, combining the Gd-based nanoparticles with 10 Gy irradiation led to significant delay of tumor growth (Miladi et al. 2015). Shi et al. designed a rattle nanoparticle with an upconversion nanoparticle core and a hollow silica shell as radiation dose amplifiers. A hypoxia-activated prodrug, tirapazamine, was loaded to overcome the oxygen dependent radiotherapy. The rattle nanoparticles had low cytotoxicity and high in vivo histocompatibility. As radiosensitizers, the upconversion nanoparticles showed significant suppression of tumor growth. In junction with tirapazamine, they were capable of killing hypoxic tumor cells through synergetic effects (Liu et al. 2015). Other inorganic nanoparticles like Y_2_O_3_ or ZnFe_2_O_3_ are undergoing investigations for their potential in radiotherapy (Meidanchi et al. 2015; Scaffidi et al. 2011).

### Reduction of side effects through nanomedicine

Reduction of side effects can be achieved by decreasing distribution of radiosensitizers or radioisotopes in normal tissues and by controlling the release of these radiotherapeutic agents (Torchilin 2001; Win and Feng 2005). The side effects of radiotherapy are often caused by unexpected damage to normal tissue. By using radiosensitizers, there are additive and synergistic advantages to the tumoricidal effect of radiation. Therefore, application of radiosensitizers will allow lower doses of radiation to attain the same/better efficiency of killing tumors. However, the unspecific biodistribution of radiosensitizers will lead to toxicity to normal tissues. The same thing applies to radioisotopes, whose accumulation in normal tissues will cause direct injury. Nanoparticles were shown to have less penetration to normal vasculature and capillaries in various parts of the body, such as the skin, lung, and heart (Eblan and Wang 2013; Sanhai et al. 2008). Therefore, controlled and sustained release of nanoparticles into the tissue prolonged exposure to the agents, which is associated with a better effect and higher tolerance for normal tissues. This was demonstrated with the clinical use of Doxil, which dramatically reduced the cardiotoxicity of doxorubicin, without compromising its anti-tumor effect (Barenholz 2012). Moreover, through chemical binding between nanoparticles and radiotherapeutic agents, the release can only occur under certain circumstances. It can either respond to the tumor microenvironment such as a low pH, redox or enzymes; or respond to an external stimuli’s like temperature change or a magnetic field (Wang et al. 2014). Such strategies dramatically decrease the release of the agents in blood vessels or normal tissues, thereby potentially limiting the side effects.

## Application of nanotechnology to combining radiotherapy with other therapies

The combination of chemotherapy and radiotherapy is one of the most effective ways to improve clinical treatment of locally advanced cancers. The concept was proposed after the discovery of fluorouracil. The concurrent chemoradiotherapy outperforms sequential therapies because chemotherapy sensitizes the tumor cells to radiation-induced killing and treatment; meanwhile the concurrent therapy avoids the repopulation of cancer cells which will occur during the course of sequential treatment (Lawrence et al. 2014). However, the increased toxicity, which is the price to pay for the synergism, becomes the main shortcoming of the strategy and is the limiting factor in its application in clinical trials.

**Fig. 2:**
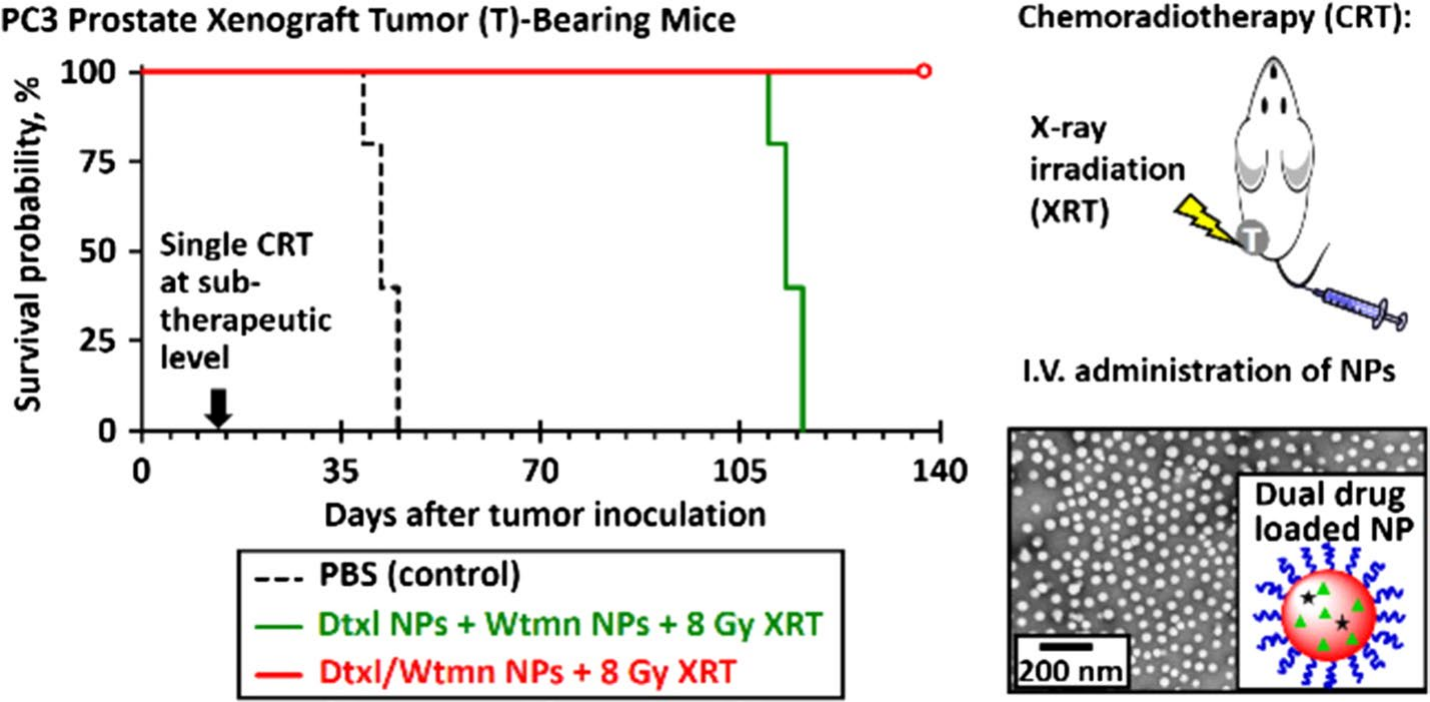
Improving cancer chemoradiotherapy treatment by dual controlled release of wortmannin and docetaxel in polymeric nanoparticles (Au et al. 2015a, b)

Nanotechnology can facilitate the chemoradiotherapy in two ways. One is to deliver chemotherapeutics by nanoparticles combined with external irradiation for combination therapy due to the radiosensitizing effect of some chemotherapeutic drugs, such as cisplatin, doxorubicin, and paclitaxel (Jung et al. 2012; Werner et al. 2013; Xiong et al. 2015). Second is to co-deliver both chemotherapeutics and radiosensitizers/radioisotopes in the same nanoparticle, which achieves the simultaneous delivery of agents at lesion as well as concise ratio control. Both nanotechnology approaches benefit from decreased toxicity in normal tissues and preferential accumulation in tumors due to the reasons mentioned previously. For instance, cisplatin is often used both as a chemotherapeutic agent and a radiosensitizer. Shi et al. reported delivery of cisplatin with a rattled-structured upconversion nanoparticle for chemoradiotherapy. The experiment was conducted on mice bearing Hela xenograft tumors. The enhanced chemoradiotherapy was achieved due to both the release of cisplatin and the high-Z metal ions (Yb^3+^, Gd^3+^) in the upconversion nanoparticles (Fan et al. 2013). Li et al. reported the combination therapy using cyclopamine encapsulated in a liquid-lipid nanoparticle system and lutetium-177-labeled core-crosslinked polymeric micelles. In 4T1 xenograft tumor model, the tumor volume was significantly smaller than monotherapy group at day 16 after treatment. The same result was also observed in Miapaca-2 xenograft tumor model. (You et al. 2015). In another study (Fig. 2), the authors showed that combining both docetaxel and wortmannin in PLGA nanoparticles changed the physiological properties in comparison to administrating each drug alone. The in vivo toxicity profile of the nanoparticles containing both docetaxel and wortmannin indicates the reduction of both hepatotoxicity and hematologic toxicity. Meanwhile, they achieved better chemoradiotherapeutic effect than each single-drug-loaded nanoparticle and combination of both single-drug-loaded nanoparticles using xenograft models (Au et al. 2015a, b).

Targeted nanoparticles are also developed for chemoradiotherapy. The cell penetration efficiency of nanoparticles can be boosted by surface modification of targeted ligand, such as folate, RGD peptide or transferrin (Mi et al. 2011a, b, 2013; Zhao et al. 2012). In one study, docetaxel-loaded, folate-conjugated nanoparticles were developed as radiosensitizers. In vivo results revealed that targeted nanoparticles were more efficient than the nanoparticles without targeting ligands. In addition, the radiosensitization efficacy was dependent on the timing of irradiation. Due to the controlled release pattern of nanoparticles, the maximal radiosensitization was different from the free radiosensitizers and should be considered carefully (Werner et al. 2011a, b). Folate-targeted nanoparticles were also applied in co-delivery of both chemotherapeutics and radioisotopes for radiochemotherapy. Paclitaxel and yttrium-90 were used for the combination therapy. The superiority of the targeted group was shown in a murine model with peritoneal metastasis of ovarian cancer (Werner et al. 2011a, b). A similar design with aptamer as the targeting ligand was developed for combining docetaxel and indium-111 and yttrium-90 (Wang et al. 2010).

In spite of the improvement of radiotherapy through nanotherapeutics, cancer cells still struggle to resist radiotherapy. Radioresistance may occur through many mechanisms such as expression of DNA repair enzymes and anti-apoptotic proteins (Al-Dimassi et al. 2014; Zhao et al. 2013a, b). Hypoxia is a key change of the tumor microenvironment after irradiation and is considered as one of the central factors leading to resistance of radiotherapy. The rapid proliferation of cancer cells and the abnormality of tumor vasculature cause the hypoxic environment around the tumor. The average oxygen partial pressure in tumors is 8–10 mmHg or 1.1–1.3%, while in other tumor tissues the average oxygen partial pressure is 35 mmHg or 4.6%. Also, it upregulates hypoxia-inducible factor (HIF), which is considered to be associated with the failure of radiotherapy (Brizel et al. 1997; Koukourakis et al. 2006; Moeller et al. 2004). It has been showed that cancer cells in the hypoxic environment are two to threefolds more radioresistant than cells under normal oxygen supply (Barker et al. 2015; Willers et al. 2013). The disease-free survival was higher in head and neck cancer patients who had pre-treatment median oxygen tensions of more than 10 mmHg compared with their counterparts in the group of less than 10 mmHg (78 versus 22%) (Brizel et al. 1997).

By targeting the signal pathways and downregulating the related genes, radiotherapy resistance can be immensely reduced or even completely negated. For example, downregulation of vascular endothelial growth factor (VEGF) helps to normalize the vasculature for reduction of hypoxia and increase radiotherapy response (Carmeliet and Jain 2011). Previous study indicated that administration of bevacizumab 48 h before radiotherapy led to synergistic effects on tumor-bearing mice models due to the transient normalization of tumor vasculature, leading to the temporary tumor re-oxygenation and improvement of radiotherapy sensitivity (McGee et al. 2010). One side effect of this approach is that it might decrease the accumulation of agents led by EPR effect, because it normalizes the tumor vasculature and decreases its leaky degree. In addition, the fibrotic process, induced by the inflammatory response after radiation, limits the eradication of tumor cells. Hence, the inhibition of TGFβ might control this process to enhance the efficacy of radiotherapy (Barcellos-Hoff et al. 1994).

Another promising approach to overcome radiation resistance is using small interfering RNA (siRNA) to target related pathways. siRNA is a double-stranded RNA with 21–23 nucleotides, which functions as the post-transcriptional regulator by cleaving targeting mRNA for a reduction of corresponding protein expression. By screening an siRNA library targeting all protein kinases and E3 ubiquitin ligases in the human genome, TRAF2 (TNF receptor-associated factor 2) was recognized as an effective target for siRNA silencing, which resulted in growth suppression of glioblastoma cells and sensitization of these radioresistant cells to radiotherapy (Zheng et al. 2008). However, the clinical use of siRNA is hindered by its sensitivity to enzymatic degradation, fast clearance, immunogenicity and incapability of entering cells (Zhao and Feng 2015). Therefore, formulation of siRNA into nanoparticles is a practical way to achieve the function of siRNA. Co-delivery of radiotherapy agents and siRNA that relates to the resistance mechanism in the nanoparticles could effectively reduce the resistance of radiotherapy and achieve synergistic effects. For example, Zhang et al. reported a nanoparticle-based siRNA delivery system composed of iron oxide nanoparticles coated with PEG and PEI. SiApe1 was delivered by this system to increase DNA deterioration after irradiation. The expression of Ape1 was knocked down over 75% in medulloblastoma cells and ependymoma cells, leading to more than threefold reduction of LD50 by irradiation in vitro (Kievit et al. 2015). Kjems et al. delivered siTNFα by chitosan/siRNA complex and completely prevented the radiation-induced fibrosis in CDF1 mice after a single dose of 45 Gy (Nawroth et al. 2010). Gao et al. used PEG-PEI copolymer for complexity of siRNA against the sCLU protein. The cell survival of MCF-7 was 38% at 0.5 Gy and 3% at 3 Gy for the combination group, compared with 93% at 0.5 Gy and 54% at 3 Gy for the exclusive radiotherapy group (Sutton et al. 2006).

## Application of nanotechnology to image-guided radiotherapy

Image-guided radiotherapy (IGRT) is the use of imaging technology for a more precise and accurate irradiation, at the tumor site instead of the surrounding tissues, during the course of radiotherapy. Computed tomography (CT), magnetic resonance imaging (MRI), ultrasound (US) and X-ray imaging are often used for IGRT.

**Fig. 3:**
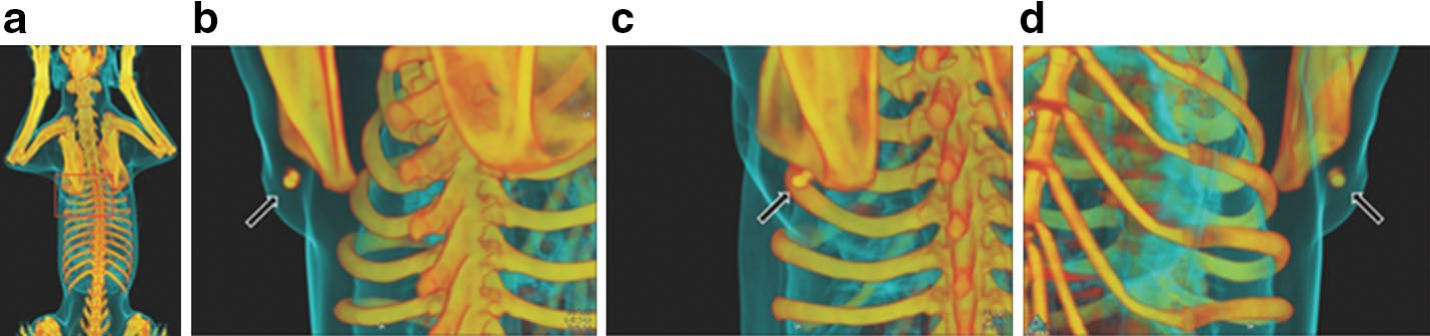
3D reconstruction based on CT images of canine patient with injected nanogel. Nanogel composed of SAIB/EtOH/PLA (75:20:5) + 30 mg PNIPAM-coated AuNPs mL − 1 administered intratumorally into a canine suffering from an intermediate-grade subcutaneous mast cell tumor (maximum distance (x × y × z); 1.82 × 5.81 × 5.32 cm^3^, CT volume; 31.64 cm^3^) adherent to the underlying soft tissue located over the dorsal aspect of the left shoulder blade. **a** Full-body scan of the canine. Area of interest indicated with a *red box*. **b**–**d** Nanogel visualized from different angles (Jolck et al. 2015)

In preclinical study, gold nanoparticles are widely used as signal enhancer for CT-guided radiotherapy. The unique physical properties of gold nanoparticles render many applications in cancer treatment, such as radiosensitizers, and agents for photodynamic therapy or photothermal therapy (Dykman and Khlebtsov 2012; Zhang 2015). Therefore, when they were used for IGRT, the theranostics is often achieved. Andresen et al. developed poly(*N*-isopropyl acrylamide) (PNIPAM)-coated gold nanoparticles in a gel matrix of sucrose acetate isobutyrate (SAIB)/EtOH/PLA as liquid fiducial tissue marker for 2D X-ray visualization (Fig. 3). The nanogel was assessed in immunocompetent mice by subcutaneous injection, which showed high-resolution micro-CT images. Its use in IGRT was examined in a canine cancer patient with a large spontaneous solid tumor. It provided enhanced image contrast for both CT and 2D X-ray imaging and was not affected by the external irradiation. No side effects were found in neither the mice model nor the canine patient (Jolck et al. 2015). In a mice model with intracerebral malignant gliomas, gold nanoparticles, 11 nm in size, were injected intravenously and IGRT was initiated by micro-CT. The uptake of gold nanoparticles was 19-fold higher in tumors than that in the normal brain. Fifty percent of mice receiving 30 Gy irradiation with gold nanoparticles showed tumor-free survival, while no mice in the exclusive radiation group survived (Hainfeld et al. 2013).

There are also researchers using composite nanoparticles for enhanced multimodal imaging or theranostic applications. One example is the nanocomposite that contains both SPIO nanoparticles and gold nanoparticles. CT, for instance, has the advantages in rapid scanning with high spatial resolution but suffers from poor soft-tissue contrast; whereas MRI gives high soft-tissue contrast but suffers from long scanning time and sub-optimal geometrical accuracy. Multimodal imaging could supply more evidence and information for optimal guidance of radiotherapy. Tsourkas et al. reported a PCL-PEG micelle system loading with both SPIO and gold nanoparticles. It realized selective tumoral accumulation and enhanced MRI of tumor margins in tumor-bearing mice. The 90-day survival rate was improved from 25 to 75% after using the nanoparticles (McQuade et al. 2015). Shi et al. synthesized a SPIO core/gold shell nanocomposite. The photothermal effect was shown with exposure to an NIR laser and a high efficiency as MRI contrast agent was ensured, indicating it as an MRI-guided theranostic platform (Dong et al. 2011).

In addition to the therapeutic and diagnostic applications, nanoparticles may also be utilized during the treatment for the monitoring and evaluation. Radiation dose is the first concern during the therapy. In situ observation of radiation dose can help to assess the therapeutic levels efficiently. Delivery dose can be confirmed by measurement of entry, exit or luminal dose, a process called in vivo dosimetry. Rege’s group developed liquid surfactant-templated formation of colored dispersions of gold nanoparticles as a facile, visual, and quantitative indicator for radiation dosimetry. This nanosensor can detect radiation dose from 0.5 to 2 Gy in a linear range. The detection range can also be expanded to 5–37 Gy through modulating the concentration and chemistry of the templating liquid surfactant. With the help of this nanosensor, the qualitative detection of radiation can be observed by naked eye, and the quantitative radiation dose can be analyzed by an absorbance spectrophotometer (Pushpavanam et al. 2015).

Radiation resistance is another issue, which is related to the hypoxic tumor microenvironment. The detection of hypoxia is necessary and instructive to determine the subsequent treatment after a primary therapy of radiation. Researchers have reported a nanosensor for hypoxia imaging which consisted of upconversion nanoparticles and an oxygen indicator. The nanosensor detected hypoxia with high penetration depth in vivo due to its near-infrared excitation and far-infrared emission. It can be reversibly quenched or illuminated under hyperoxic or hypoxic conditions with a high signal-to-noise ratio. It presented substantially enhanced sensitivity, high selectivity, and high specificity for the detection of oxygen changes in hypoxic environment (Liu et al. 2014).

Management of side effects also plays a critical role in radiotherapy monitoring, especially the vascular injury. Patients receiving radiotherapy for breast cancer are four times more likely to suffer cardiovascular events (Baskar et al. 2012). The long-term cardiovascular side effects include myocardial infarction, atherosclerosis, and stroke (Aleman et al. 2003; Russell et al. 2009). The reason for these cardiovascular events, caused by radiotherapy, may relate to acute up-regulation of proinflammatory cytokines and adhesion molecules at the endothelium of injured blood vessels (Halle et al. 2010). Wang et al. developed a basement membrane (BM)-targeting nanoparticle to directly observe this injury. The nanoparticles comprised a synthetic peptide targeting collagen IV fiber, which enables the nanoparticle to bind to the collagen IV-rich BM on the site of endothelium damage. Its ability to identify an early-stage blood vessel injury induced by high-dose radiotherapy was demonstrated in a Murine model (Au et al. 2015a, b; Kamaly et al. 2013).

## Clinical trials in translation of nanotechnology to radiotherapy

Clinically, the liposomal doxorubicin (Caelyx) plus conventionally fractionated radiotherapy was the first reported clinical trial for locally advanced non-small-cell lung cancer (NSCLC) along with head and neck cancer (Koukourakis et al. 1999). It achieved 40% complete response and 87% partial response but a grade 3 esophagitis for the patients with stage IIIb NSCLC (Koukourakis et al. 2002). Liposomal cisplatin concurrent with conventionally fractionated radiotherapy was conducted on 20 patients with head and neck cancer. Fifty-five percent of the patients had complete response at the primary tumor site but with grade 3 skin and mucosal toxicities (Rosenthal et al. 2002). Abraxane is the albumin-bound paclitaxel. Due to the nano size of the protein, this prodrug is considered as the nano-drug and approved by FDA. There are several ongoing clinical trials using paclitaxel albumin-stabilized nanoparticles (nab-paclitaxel) for chemoradiotherapy. A phase I trial combined nab-paclitaxel and carboplatin followed by chemoradiation for treatment of recurrent head and neck cancer (NCT01847326). In a phase II trial, nab-paclitaxel and gemcitabine hydrochloride were used as chemotherapeutics followed by radiotherapy in treating patients with pancreatic cancer (NCT02427841). In addition, polymer-based nanoparticles, including polymer-drug conjugates or polymeric nanoparticles, are getting into the clinic. For example, a phase I trial was conducted to determine the maximal tolerated dose of poly(l-glutamic acid)-paclitaxel and concurrent radiation for patients with esophageal and gastric cancer (Dipetrillo et al. 2006). The initial dose of paclitaxel was 40 mg/m^2^ per week, with 50.5 Gy radiation for 6 weeks. The dose was increased in 10 mg/m^2^ per week of paclitaxel. Three out of four patients showed dose limiting toxicities at 80 mg/m^2^. Four out of twelve patients with loco-regional disease had a complete clinical response. Another phase I trial combined poly (l-glutamic acid)-paclitaxel with temozolomide and concurrent radiation for high-grade gliomas, revealing severe hematologic toxicity (Jeyapalan et al. 2014). In this study, seven out of 25 patients showed grade 4 myelosuppression. Hematologic toxicity lasted up to 5 months, which indicated paclitaxel are not safe to combine with temozolomide. However, it showed 11.5 months of progression-free survival and 18 months of median overall survival, revealing that poly (l-glutamic acid)-paclitaxel combined with radiation might be efficient for treating glioblastoma. We are also conducting a phase Ib/II trial to evaluate the maximal tolerated dose of CRLX101, a nanoparticle formulation with camptothecin-cyclodextrin-PEG polymeric prodrug, when combined with neoadjuvant therapies capecitabine and radiotherapy (NCT02010567).

The application of nanotechnology in clinical imaging and diagnostics improves the contrast between tumor and bony or soft-tissue anatomy, resulting in a more competent radiotherapy treatment. Superparamagnetic iron oxide (SPIO) nanoparticles have been approved clinically to enhance the T2 contrast of MRI, such as ferumoxide, ferumoxtran-10 and ferucarbotran. The superiority of SPIO was revealed in a study for detection of clinically occult lymph node metastases. Eighty-eight patients with resectable prostate cancer were involved in an MRI scan. The sensitivity of MRI scan increased from 35.4 to 90.5% for patients with lymphotrophic SPIO nanoparticles, and the prediction for all patients with lymph node metastases was correct (Harisinghani et al. 2003). Clinically, irradiation on regional lymphatics is often applied in the curative treatment of many cancers. However, the location and treatment volume of lymph nodes are difficult to define. Therefore, MRI lymphography with SPIO can assist in radiation planning (Meijer et al. 2012; Ross et al. 2009; Vilarino-Varela et al. 2008). For example, a total of 55 patients with different forms of cancer underwent an MRI scan with ferumoxtran-10. An average of 30 lymph nodes were identified in each patient, and the distribution of nodal distance to the closest artery or vein was observed. The information provided the probability to optimize the irradiation dose on at-risk lymph nodes and normal tissues (Dinniwell et al. 2009). In addition to SPIO, gadolinium nanoparticles are also studied for the T1 contrast enhancement. AgulX nanoparticles are composed of a polysiloxane network surrounded by gadolinium. When compared with the commercially used agent on healthy animals, it displayed better MRI pictures. Furthermore, the radiotherapy guided by AgulX nanoparticles showed an increased medium survival time (Le Duc et al. 2014).

## Challenges in translation of nanotechnology to radiotherapy

Nanomedicine has emerged for decades as a promising field to address many medical problems. In clinical cancer treatment, a few products have been commercialized like Doxil or Abraxane. Instead, most of the attempts for nanoparticle-based clinical trials failed, as the efficacy is not as high as it indicates in animal models, like CALAA-01. One of the most important foundations of the field, the EPR effect, is challenged by more and more clinical data. With the deeper understanding of tumor microenvironment, it seems that the moderate increase of therapeutics by EPR effect is far from enough to cure cancer. At the same time, the long circulation time of nanoparticles might increase systemic toxicity.

However, the problem in formulation of radiotherapeutics will always exist, and the benefits from nano formulation to achieve increased solubility, controlled release and combinational delivery are obvious. Nanotechnology will still be a powerful candidate in solving many problems in radiotherapy. Instead of satisfaction or abandonment to current status of nanomedicine, more meticulous and in-depth work is necessary. Current preclinical research with animal models cannot precisely predict the therapeutic or toxic effect in patients. Correlations among in vitro, in vivo and patient results are worth to find out. The biological mechanisms revealed from the animal models provide us reference to design our nanoplatforms, which should be the primary principle instead of endless sophistication of the nanoplatforms. Comprehensive toxicity testing and understanding of the biological pathway behind it are required before moving on to clinical trials.

## Conclusions

The field of radiation oncology is constantly evolving with technological advances. These advances include delivering high doses to more conformal volumes and moving targets. However, these improvements did not necessarily result in a significant change in cure rates or local control rates achieved by radiotherapy. The most reasonable explanation is the efficacy of radiotherapy is limited by normal tissue toxicity, tumor resistance, and accurate radiotherapy delivery. Thus, radiation oncology can potentially gain from further exploring the contribution of nanotechnology to overcome these limitations.

Nanotechnology can be used to potentiate the delivery and/or concentration of radiosensitizers or radioisotopes, thus enhancing their anti-tumor activity. Moreover, recent studies directed toward the effects of radiotherapy on tumor microenvironments have given rise to other combinational treatment of radiotherapy, especially with immunotherapy. Radiotherapy leads to increased exposure and presentation of tumor antigens, which triggers inflammatory cytokine signaling and immune cell recruitment. While cancer immunotherapy, like checkpoint blockade or chimeric antigen receptor (CAR) T cell therapy, shows promising results clinically, combining both of them with nanotechnology is still under investigation.

The use of nanotechnology in imaging can also be used for adaptive radiotherapy or IGRT. Therefore, in our view, it is imperative to continue exploring the role of nanotechnology in improving the ability of radiotherapy to damage cancer cells. Nanotechnology may provide an alternative means to overcome the limitation of dose escalation (radiosensitizers, radioisotopes) and physical-technical features (IGRT) that can be manipulated to further improve treatment efficacy.

## Abbreviations

CT: computed tomography
DSPE: 1,2-distearoyl-sn-glycero-3-phosphoethanolamine
EPR: enhanced permeability and retention
EtOH: ethanol
FDA: food and drug administration
HIF: hypoxia-inducible factor
ID: injection dose
IGRT: image-guided radiotherapy
MTD: maximum tolerated dose
MRI: magnetic resonance imaging
NSCLC: non-small-cell lung cancer
PEG: polyethylene glycol
PEI: polyethylenimine
PLGA: poly(lactic-co-glycolic acid)
PNIPAM: poly(*N*-isopropyl acrylamide)
SPECT: single-photon emission computed tomography
SPIO: superparamagnetic iron oxide
RGD: arginylglycylaspartic acid
ROS: reactive oxygen species
SAIB: sucrose acetate isobutyrate
siRNA: small interfering RNA
TGF: transforming growth factor
TNF: tumor necrosis factor
TRAF2: TNF receptor-associated factor 2
US: ultrasound
VEGF: vascular endothelial growth factor
Wtmn: wortmannin
